# FTIR spectroscopy of whole cells for the monitoring of yeast apoptosis mediated by p53 over-expression and its suppression by *Nigella sativa* extracts

**DOI:** 10.1371/journal.pone.0180680

**Published:** 2017-07-12

**Authors:** Wafa Mihoubi, Emna Sahli, Ali Gargouri, Caroline Amiel

**Affiliations:** 1 Laboratoire de Biotechnologie Moléculaire des Eucaryotes, Centre de Biotechnologie de Sfax, Sfax, Tunisia; 2 Unité de Recherche Aliments Bioprocédés Toxicologie Environnements (UR ABTE) EA 4651, Université de Caen Normandie, Boulevard Maréchal Juin, Caen, France; Rush University Medical Center, UNITED STATES

## Abstract

p53 over expression in yeast results in cell death with typical markers of apoptosis such as DNA fragmentation and phosphatidylserine externalization. We aimed to substitute/supplement classical fluorescent techniques (TUNEL, Annexin V, ROS detection) usually used to detect biochemical changes occurring during yeast apoptosis mediated by p53 over expression and the effect of anti-apoptotic purified molecules from Nigel (*Nigella sativa*) extracts on these same yeasts by the label free technique of FTIR spectroscopy. The comparison of the entire IR spectra highlighted clear modifications between apoptotic p53-expressing yeasts and normal ones. More precisely, DNA damage was detected by the decrease of band intensities at 1079 and 1048 cm^-1^. While phosphatidylserine exposure was followed by the increase of νsCH_2_ and νasCH_2_ bands of unsaturated fatty acids that were exhibited at 2855 and 2926 cm^-1^, and the appearance of the C = O ester functional group band at 1740 cm^-1^. In a second step, this FTIR approach was used to estimate the effect of a purified fraction of the Nigel extract. The modulation of band intensities specific to DNA and membrane status was in agreement with apoptosis supression in presence of the Nigel extracts. FTIR spectroscopy is thus proven to be a very reliable technique to monitor the apoptotic cell death in yeast and to be used as a means of evaluating the biomolecules effect on yeast survival.

## Introduction

Fourier-transform infrared (FTIR) spectroscopy is a vibrational spectroscopic technique used to solve the chemical composition information of a sample and to provide its “molecular fingerprint” [1]. This technique has been previously explored for different measurement modes in many biological systems [2,3] to become a powerful tool for the cell components analysis, such as nucleic acids [4] proteins [5] and membranes [6]. This technique has also been useful for complex biological systems, such as tissues [7] and microorganisms [3,8,9] because of its ability to analyze both qualitative and quantitative, molecular and structural indications for samples components. FTIR spectroscopy is a non-destructive technique [10,11], that allows a rapid analysis of biochemical and structural changes (without the need for reagents) such as DNA, protein and cell membrane [10,12–14]. The analysis of spectral data provides qualitative and quantitative information of a cell component on the basis of peak’s shifts, bandwidths and band intensities. Under stress, data can simultaneously determine changes in proteins, lipids, carbohydrates and nucleic acids at the level of functional groups [10, 15]. FTIR spectroscopy was used by lot of researchers to study metabolic alterations in stressed microorganisms such as the bacterial response to antibiotics [16], the effect of starvation [17,18], and of different environmental stresses [19–24], as well as yeast alterations by chemical compounds such as ethanol [25,26]. Bellisola and Sorio [27] consider that FTIR spectroscopy in medical biology is an interesting emerging opportunity.

Apoptotic cells exhibit morphological changes including membrane blebbing, cell shrinkage and biochemical changes including chromatin condensation and DNA fragmentation [28], changes in protein synthesis [29] and translocation of phosphatidyl-serine (PS) from the cytoplasmic side of the plasma membrane to its outer side, and finally exposing PS to the medium. FTIR spectroscopy has already been used to study apoptosis [30–35], necrosis [36], cell cycle, differentiation and proliferation in different cell lines and tissues [37, 38], but never in yeast, and this is where the novelty of this research lies.

Yeast apoptosis was detected for the first time in a strain carrying mutation in the AAA-ATPase gene CDC48 [39]. Other works identified stimulants including acetic acid [40], sugars [41], hydrogen peroxide (H2O2) [42], aspirin [43], and essential oils [44]. In addition, the heterologous expression of pro-apoptotic proteins such as Bax [45] and caspase 1 and 3 in yeast leads to apoptosis [46]. Yeast apoptosis has been described to occur in different physiological scenarios [47]. Particularly, *S*. *cerevisiae* apoptosis shares several morphological and biochemical features with the mammalian one, despite some peculiar differences. Nuclear DNA fragmentation and phosphatidyl serine externalization are general common markers of both mammalian and yeast cell death. However, the characteristic feature of mammalian apoptosis is the activation of caspases “Aspartate-specific”, and proteases that initiate and achieve the cell death cascade through of the cell components degradation while yeast contains only one caspase homolog gene, called YCA1, encoding for a metacaspase which is Arginine/Lysine-specific, [48]. Several other differences do exist between both apoptosis mechanisms, due to the absence of specific genes such as bcl2, p53, bax, MDM2 in yeasts.

In a previous study our group showed that Human p53 over expression in the yeast *Saccharomyces cerevisiae* resulted in cell death with characteristic markers of apoptosis: an exposure of PS and DNA strand cleavage as shown by Annexin V staining and TUNEL (terminal-dUTP nick end labeling) assays respectively, including higher ROS production [49]. This system model was exploited to purify molecules from Nigel (*Nigella sativa*) extracts that can suppress the negative effect of the p53 expression in yeast (unpublished results).

The aim of this study was to get further insight into the capability of the non destructive technique FTIR spectroscopy to confirm the p53 apoptosis marker in yeast found previously by fluorescence technique and to investigate the role of purified anti-apoptotic fraction from Nigel (*Nigella Sativa*) extracts in the inhibition of such apoptosis.

## Materials and methods

### Strains and media

The used yeast strain is W303-1B (*MATα ade2-1; ura3-1; his3-11*,*15; leu2-31*,*12; trp1-1; can1-100*) [50] called W303. The yeast multicopy YepDP8-1 vector (provided by Denis Pompon, C.G.M., CNRS-Gif Sur Yvette, France), called here pDP, was used to express human p53 (wt form) under the control of the Gal10/Cyc1 galactose-inducible yeast promoter. This vector contains the 2 μ replication origin and the ura3 marker [51]. The insertion of the wt p53 cDNA into this yeast expression vector has been previously described [52].

Yeast minimal medium (MM): contained 0.67% yeast nitrogen base (Difco), tryptophan (40 mg/L), histidine (20 mg/L), leucine (120 mg/L) and adenine (40 mg/L). MM was supplemented with either 2% glucose (for normal growth) or 2% galactose (for p53 gene induction) as carbon sources and called respectively MMGLU (repression of p53 expression by glucose) and MMGAL (induction of p53 expression by galactose). The growth kinetics were performed on liquid and solid media.

### Cell growth kinetics

Yeast culture of W303/pDP and W303/p53 was grown on glucose-containing MM (MMGLU) until stationary phase (O.D = 4units). 400μL from MMGLU cultures of W303/pDP and W303/p53 were inoculated in MMGAL media to induce P53 expression (W303/p53) and in MMGAL added with the active fraction from Nigel extract. Yeasts were cultured overnight under continuous agitation (150 rpm) at 30°C.

### Apoptosis assessment by Annexin V-FITC/PI staining

Exposure of phosphatidylserine and internal PI staining were detected using the Annexin V Staining kit (life science), essentially as described by Madeo et al. (1997) [53].

Yeast cells were harvested by 4000rpm, washed three times with 1.2 M Sorbitol, 100 mM KH_2_PO_4_, 50 mM citric acid. Thereafter, the spheroplasts were obtained by incubation in lysis buffer (1.2 M Sorbitol, 100 mM KH_2_PO_4_, 50 mM citric acid, 0.2% β-mercaptoethanol, and 0.2 mg/mL (zymolyase) for 1 h at 37°C. Spheroplasts were washed with 1.2 M Sorbitol, 100 mM KH_2_PO_4_, 50 mM citric acid, and suspended in the binding buffer (1.2 sorbitol, 10 mM HEPES pH 7.4, 140 mM NaCl, 2.5 mM CaCl_2_). Spheroplastes were stained with 10μL ofAnnexin V 15 min in darkness at room temperature, washed with binding buffer, then stained with 10μL of PI., samples were immediately analyzed in the cytometer.

### Flow cytometry analysis

A cyflow^®^Space flow cytometer (Sysmex-Partec) equipped with a 488 argon laser was used to measure single cell fluorescence. Annexin fluorescence was measured using the green 536±25 nm band pass filter (FL1). Fluorescence of PI was measured using the orange 590±25 nm band pass filter (FL2).

Fluorescent beads of 10 μm in diameter were added in order to normalize the flow cytometer settings. The voltage for forward scatter (FSC) and side scatter SSC were fixed at 120 and 200, respectively. The flow rate was adjusted to 120 μL/ min. Data were collected for 10.000 events using logarithmic amplification. Unstained cells were used as controls and each assay was run in duplicate. Data analysis was investigated using winlist software (verity software, USA).

### FTIR spectroscopy

Cultures were stopped after 32 h and harvested by centrifugation (7000 rpm, 10 min). The Pellets were washed twice with saline solution (0.7% NaCl) and re-suspended in 1 mL of the saline solution. Five microlitres of the concentrated yeast were deposited on a selenium multi-plate (96 wells) in 3 replicates and dried in a desiccator under1.5 bar. FTIR measurements were performed in transmission mode using HTS-XT Tensor 27 spectrophotometer (Bruker, Marne la vallée, France). Each experiment was replicated from 3 independent cultures, in order to optimize and control reproducibility [54].

The spectra were registered using 64 scans, at a resolution of 4 cm^-1^ baseline corrected and normalized using OPUS 6.5 (Bruker) software as previously described in [55]. An average spectrum was then calculated for each independent culture. If some spectra did not pass the ‘‘quality test” and spectrum reproducibility, 3 more independent cultures were performed to obtain 3 spectra per strain. Spectra analysis would be performed using OPUS 6.5 software (Bruker) by hierarchical cluster analysis (HCA) peaks measurements and integration. To verify the similitude between spectrums recorded from the same culture, calculation of the similitude percentage was performed in the whole spectra using search standard program: similitudes have to be more than 99% [54]

### Plant extracts preparation

Nigel seeds were crushed in a blender (Rutschmuhle) to obtain powdered material. A volume of 30 mL of distilled water was added to 10 g of the powder [56]. Following continuous homogenization into a polytron apparatus (Kinematica-Gmbh), the mixture was centrifuged at 10.000 rpm for 15 min. The supernatant (of brownish orange color) was filter-sterilized on 0.45 μm filter. Clarified solutions were stored in sterilized tubes at 4°C until chromatographic separation.

### Preparative High Performance Liquid Chromatography (HPLC)

A preparative HPLC Knaeur system-model 1100 consisting of preparative pump wellchem K-1800 and a UV detector Eurochrom K-2501, using a PL Aquagel OH-40-10μm (300*25mm) with protein separation range from 10 to 200 kDa was used. The plant extract was filtered on 0.45 μM and injected. The fractions were eluted at a flow rate of 5 mL/min and UV monitored at 280 nm, using an isocratic phase consisting of water. Eluted fractions were dried then dissolved in water.

### Preparative Fast-Performance Liquid Chromatography (FPLC)

A preparative FPLC Biorad system made up of 2128 model using anionic unoQ12. Active G1 fraction was filtered through 0.45 μm, deposited on an anionic column at pH 5 (citrate buffer), with a flow rate of 3 mL/min and a UV monitoring at 280nm.

The elution gradient consists of: a- isocratic flow with 100% A-buffer (citric buffer/tris buffer) for 3 min, b- a sample injection, c- isocratic flow with 100% A-buffer for 20 min, linear gradient with 100% B-buffer (composed of 1 M NaCl in A-buffer), d- isocratic flow with 100% B-buffer for 5 min, e- isocratic flow with 100% A-buffer for 5 min.

### Analytical High-Performance Liquid Chromatography (HPLC)

An HPLC Agilent system consisting of model Agilent 1100 and equipped with a “C-18 reversed phase” column (Eurosphere 100–5 C-18” 250×8mm TC 01107) was used. Active Q1 fraction was firstly filtered through 0.45μm, deposited on the column and fractions were eluted at a flow rate of 0.5 mL/min and a UV monitored at 260 nm and by RID (refractive index detector).

The elution gradient, used for the reversed phase C-18, was a linear one and consisted of solvent A (1% formic acid) and solvent B (100% methanol). The solvent gradient started at 95% A and 5% B, reaching 75% A at 10 min, 65% A at 30 min, 55% A at 35 min, 55% A at 40 min, 50% A at 45 min, 45% A at 50 min, 30% A at 53 min, 25% A at 56 min, and 20% A at 60 min, followed by a post-time isocratic plateau for 10 min at 95% A before the next injection. The flow rate was 0.5 mL/min.

### Northern blotting

Northern blot were performed as described by Sambrook et al. (1989) [57] using Hybond N membranes (Amersham). Formamdehyde gel was performed using 20 μg of RNA for each sample. Hybridization was performed with an [α-^32^P] radio-labelled 650 bp fragment of cDNA p53.

## Results

### p53 mediated yeast apoptosis detection by fluorescent methods

Previous studies proved that the human p53 over-expression, under the control of a tightly regulated galactose-inducible promoter, induces yeast cell death with characteristic markers of apoptosis: exposure of phosphatidyl-serine and DNA strand cleavage as shown by Annexin V staining and TUNEL (terminal dUTP nick end labeling) assays respectively [49].

**Fig 1:**
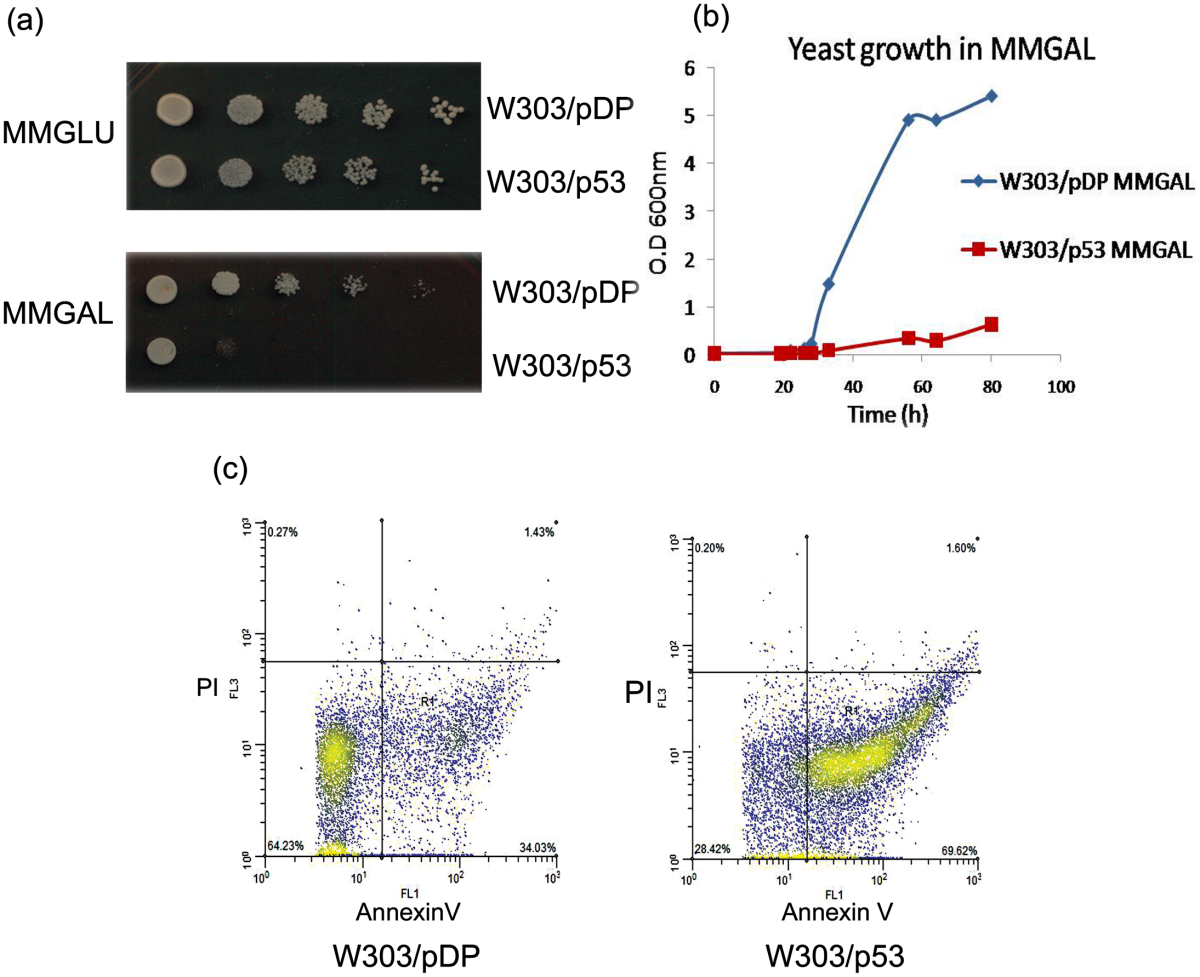
Effect of p53 expression on yeast growth of the recombinant yeast clones W303/pDP and W303/p53. (a) Recombinant yeast clones W303/pDP and W303/p53 growth on solid (MMGLU media serves as a control of cell number) and (b) on liquid media. (c) flow cytometric analysis of the phosphatidylserines externalization by FITC-Annexin V/PI costained W303/pDP and W303/p53.

Fig 1 shows the inhibition of yeast growth of W303/wt p53 and control W303/pDP cells under inducible condition on MMGAL (Fig 1a). This result was confirmed by the growth kinetic in the MMGAL liquid (Fig 1b).

Apoptosis was assessed by flow cytometry analysis of phosphatidylserine externalized at the outer phase of the cytoplasmic membrane, a phenomenon that was considered an early event of cell death and was detected by labeling cells with fluorescein coupled annexin V (green dye) PI was used to distinguish apoptosis from necrosis. Cell population stained Annexin +/pI- is considered apoptotic. The percentage of apoptoctic cells increases from 34.03% in W303/pDP stain (control) to 69.62% in W303/p53 (Fig 1c).

### FTIR analysis of p53 expression in *S*. *cerevisiae*

We planned here to harness the FTIR spectroscopy in the study of apoptosis in such lower eukaryote and evaluate the effect of an anti-apoptotic bio-molecule on this phenomenon. The originality of our study stems from the fact that FTIR spectroscopy is used here for the first time to study molecular changes in yeast apoptosis mediated by the human P53.

**Fig 2:**
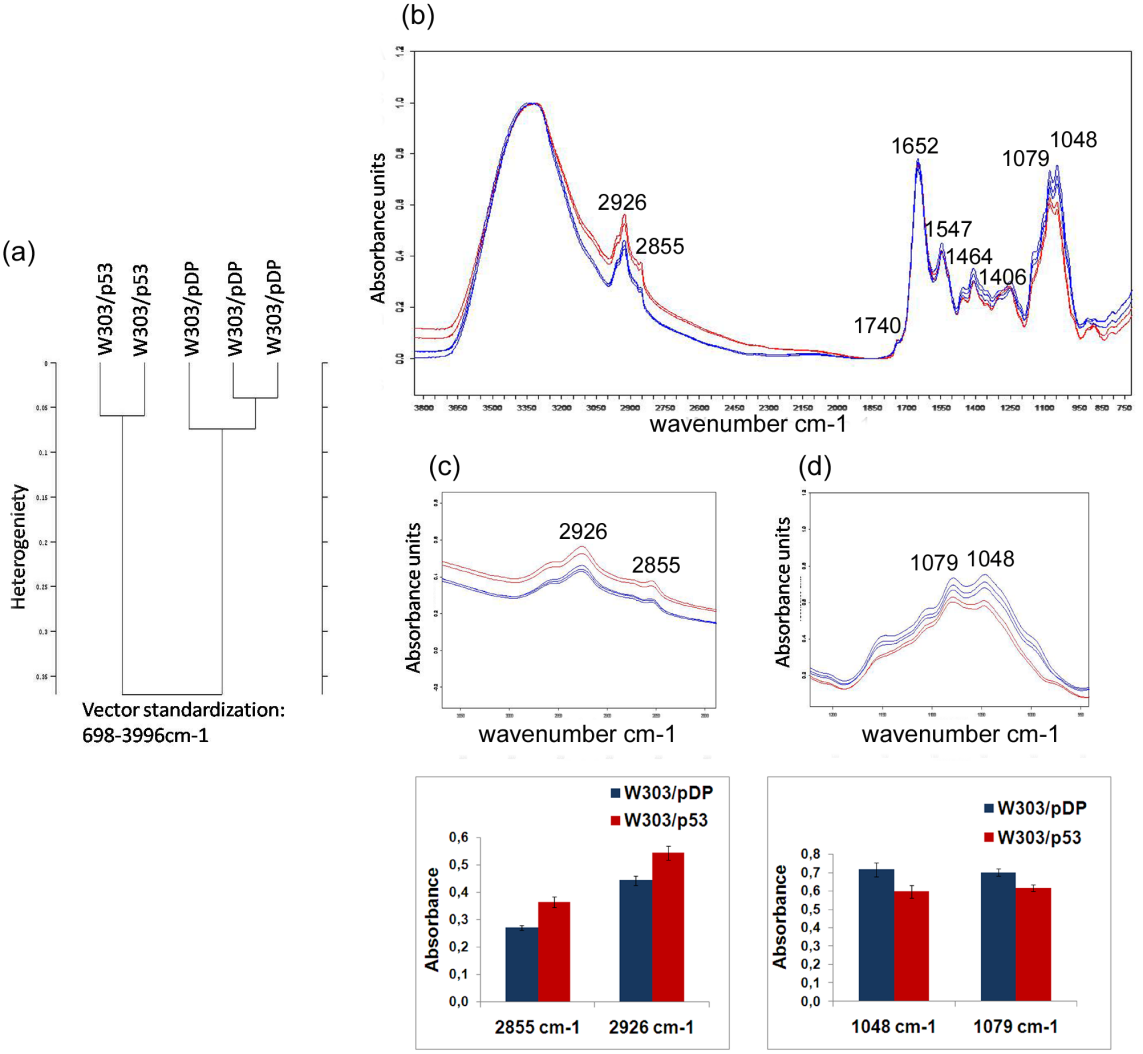
FTIR spectroscopy analysis of the whole recombinant yeast clones W303/pDP and W303/p53. (a) Dendrogram representing hierarchical cluster analysis of W303/pDP and W303/p53 FTIR spectra. (b) FTIR spectra of W303/pDP (blue line) and W303/p53 (red line) grown on MMGAL, (c) spectral intensity band changes in 3000–2820cm^−1^ region corresponding to membrane changes and spectral intensity band changes in 1187-945cm^-1^ region showing DNA changes (d); the values are means of three independent experiments.

Fig 2b depicts the normalized FTIR spectra of the transformed yeast cells W303/pDP (control) and W303/p53 that are growing under inducible conditions on MMGAL (Fig 1b).

FTIR spectra of normal cells (W303/pDP) and apoptotic cells (W303/p53) were compared using Hierarchical cluster analysis (HCA) (Fig 2a). Spectral apoptosis induced by P53-overexpression markers were sought through direct observations of its IR spectrum (Fig 2b); Peak assignments are based on previous FTIR studies in cell apoptosis as described in Table 1.

**Table 1:** Spectral peak assignments.

Wave numbers (cm^-1^)	Spectral peak assignment
1048	νC-O of polysaccharides nucleic acids
1079	νC-O-C ofnucleic acids and phospholipids that indicates the degree of oxydative damage in DNA
1406	Carboxylates of amino acids and δSCH_2_ of lipids.
1464	δSCH_3_
1547	Amide II: proteins, δN-H (bending) and νC-N (stretching)
1652	Amide I: proteins, C = O stretching
1740	νC = O ester of lipids
2855	ν_s_CH_2_ (lipids)
2926	ν_as_CH_2_ (lipids)

The results are expressed as the mean ± standard deviation (SD). The statistical significance of the differences was evaluated by Student's t-test; P<0.05 which was considered to indicate a statistically significant differences.

### The 3000–2820cm−1 region

Absorptions in this region are principally assigned to the symmetric and asymmetric stretching modes of methylene and methyl groups of lipids [58]. The intensities of bands 2855 cm^-1^ and 2925 cm^-1^ correspond respectively to CH_2_ symmetric stretching (ν_s_CH_2_) and CH_2_ asymmetric stretching (ν_as_CH_2_). Compared to the control strain (W303/pDP), the band intensities at 2855 and 2925 cm^-1^ increased from 0.270±0.009 and 0.443±0.017 to 0.364±0.020 and 0.544±0.026 in apoptotic W303/p53 cells (Fig 2c). This result was confirmed by the peak integration of 2993 to 2855 cm^-1^ area (data not shown) that increased from 14.843±0.983 in controls to 16.077±0.845 in apoptosis.

### 1758-1477cm^-1^ region

This region is dominated by the amide I and amide II bands, characterized by absorption at 1652 and 1547 cm^-1^, respectively. This originates from the vibrations of the amide groups (COONH) of proteins, and is often an indicator of their secondary structure.

In our study, there are no changes in amide I and amide II intensity bands. In addition amide I/amide II intensity ratio had no conformational changes of membrane proteins.

However a weak band at 1740 cm^-1^ appears in apoptotic W303/p53, that arises from C = O stretching vibrations of the ester functional groups in lipids [34].

### 1187-945cm^-1^ region

Band changes in this region are associated with mainly macromolecules like carbohydrates and polysaccharides that are related to the amount of nucleic acids, the 1048 cm^-1^ band is attributed to the νC-O stretching [59] of nucleic acids: desoxyribose [30, 36], RNA and ribose [35]. The absorption band at 1079 cm^−1^ corresponds to the symmetric PO_2_ (νsPO_2_−) stretching vibrations of the phosphate groups of the DNA double stranded backbone [60,37,61], the νC–O stretching vibrations of nucleic acid polysaccharides, and the νC–O–P stretching vibration of phosphorylated lipids [35,36]. Therefore, the 1079 cm^-1^ absorption band is reported to reflect the cell DNA content [62] and this indicates the DNA oxidative damage degree [30].

In this region, the most prominent differences feature between W303/pDP and the W303p53 apoptotic cells are the bands decreased intensities at 1048 and 1079 cm^-1^ (Fig 2d) which indicates a loss of nucleic acids content, reflecting a DNA damaged state. This finding corroborates the previous results of DNA fragmentation analyzed by flow-cytometry in p53-mediated yeast apoptosis [49]

### The anti apoptotic effect of Nigel fraction

The current yeast genetic system was used for the screening of natural molecules having a protective effect against p53-mediated apoptosis in yeast. Nigel (*Nigella sativa*) seeds were chosen as starting materials, as they are highly considered for their richness in antioxidant compounds.

**Fig 3:**
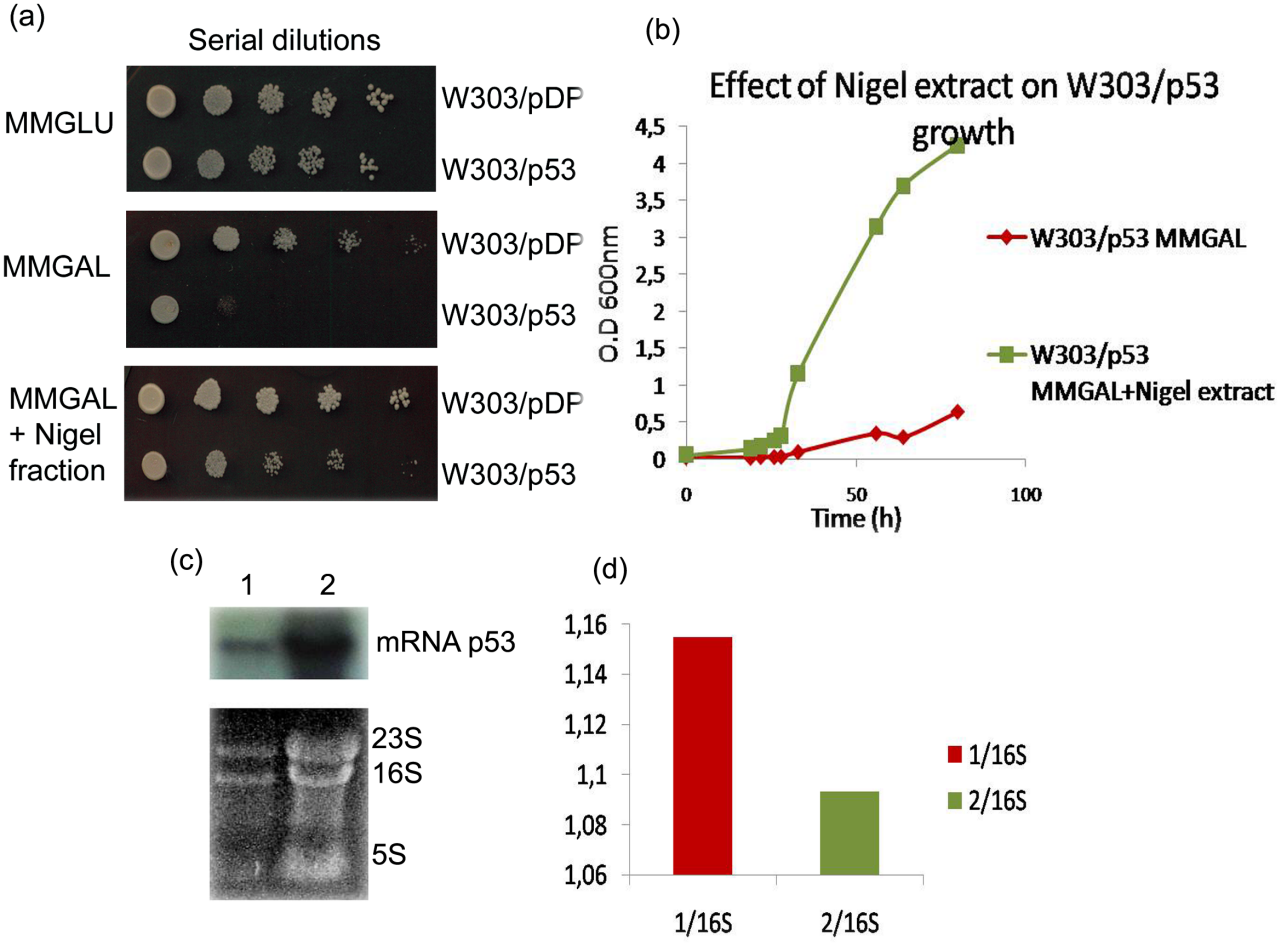
Effect of purified fraction from Nigel extract on the yeast growth of W303/p53. (a) Yeast clone W303/p53 growth on solid and (b) on liquid media MMGAL in absence and presence of purified Nigel extract. And (c) northern blot analysis of p53 mRNA level in control cell W303/p53 in inducible condition in absence (1) and in presence of Nigel extract (2).(d) Quantification of the blot.

Fig 3a shows that, in the presence of 4 mg dry weight of the Nigel extract that has been spread on the galactose plates, the growth of p53^+^ yeast cells was restored (Fig 3a). Conversely in the absence of the plant extracts, as expected, a great difference was observed between the control and the p53 expressing cells: while the growth of W303/pDP cells was normal, that of W303/p53 was strongly affected (Fig 3a).

In order to check the results obtained on solid media, the growth of cells was tracked in liquid MMGAL media with or without addition of Nigel extract. As expected, in the absence of extracts, the growth of W303/p53 was inhibited (Fig 3b). When plant extracts were added to the medium culture, the growth of p53+ cells was almost completely restored (Fig 3b). Nigel extract can act on the p53 expression itself or on the resulting effect of p53 expression, so that a northern blot was performed to study transcriptional level of p53 in presence of Nigel extract. Fig 3c shows clearly the presence of mRNA p53 in W303/p53 grown in MMGAL in presence of Nigel extract, which mean that Nigel extract did not affect p53 expression.

Guided fractionation was applied using many chromatographic columns as described in the materials and methods part, and there was just one active fraction responsible for the anti apoptotic effect.

### FTIR study of the anti apoptotic effect of Nigel fraction extract

The beneficial effect of Nigel fraction was shown either on MMGAL solid or liquid media. In this part, we investigated the contribution of the FTIR technique to study the effect of Nigel fraction on apoptosis features in W303/p53 recombinant yeast.

**Fig 4:**
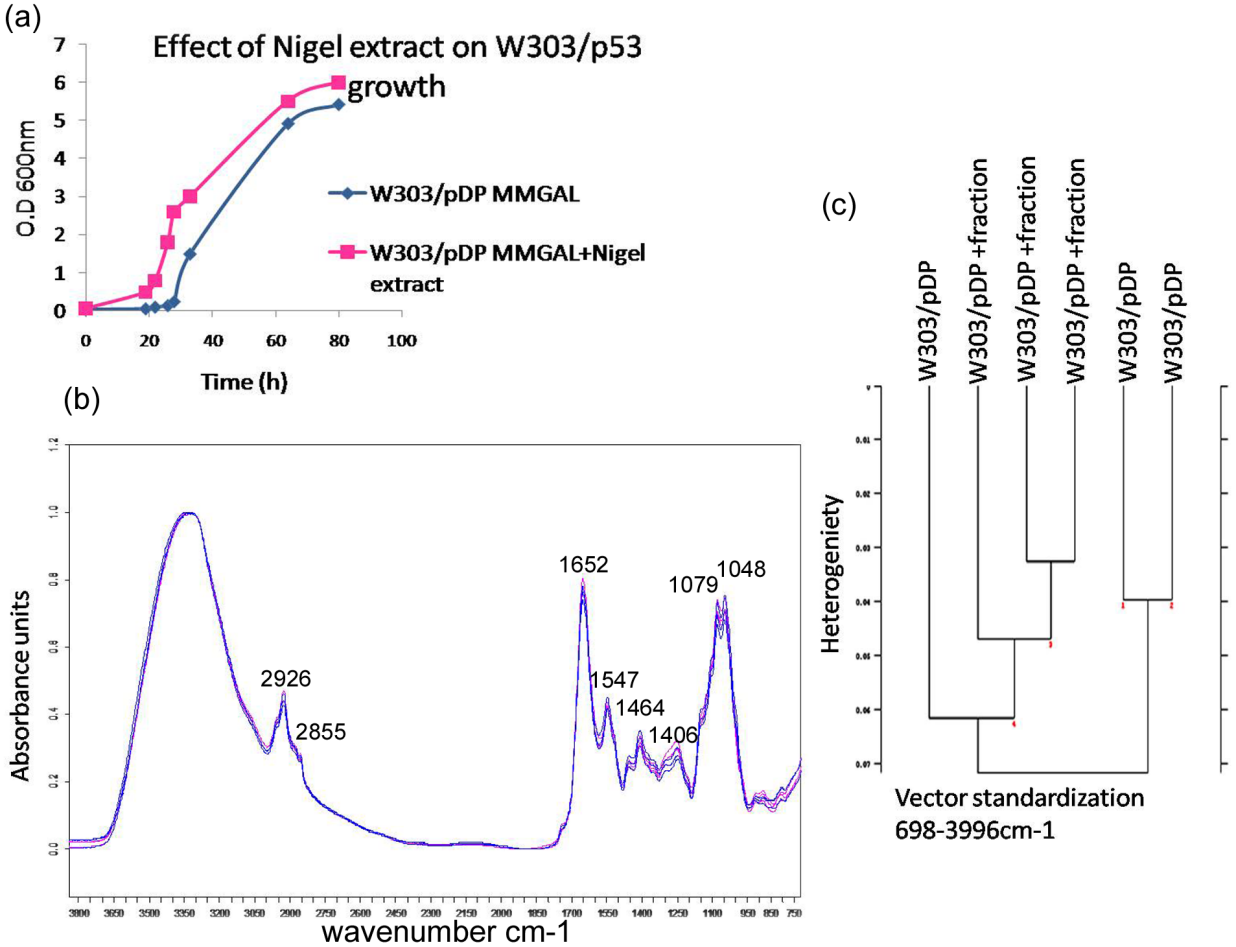
(a) Growth kinetics of W303/pDP on liquid MMGAL in presence or absence of nigel fraction. (b) FTIR spectra of W303/pDP grown on MMGAL in absence (blue line) and in presence (purple line) of Nigel fraction (c) Hierarchical Cluster Analysis of infrared spectra of W303/pDP obtained from cultures with or without nigel fraction.

In order to verify that Nigel fraction has no effect on yeast growth, FTIR spectra of control cells W303/pDP grown in MMGAL with and without the active Nigel fraction were analyzed and showed no significant difference between both spectra (Fig 4a). Moreover, the spectra cluster analysis shows that there are no spectral conformational changes (Fig 4c). It is known that FTIR spectra can be divided into spectral regions where strong absorption is related to specific component. These spectral regions include 3050-2800cm-1 where fatty acids predominates, 1750-1500cm-1 that ascribed to amide bands of proteins and peptides, 1500-1250cm-1 for carboxylic acids, and 1200-900cm-1 the polysaccharide region define the cell metabolic fingerprint. Since all these regions of the two spectra superimpose perfectly, we conclude that there would be no effect of the Nigel extract on cellular metabolism yeast cells (Fig 4).

### 3000–2820cm^−1^region

Concerning W303/p53, Fig 5 shows that in the presence of the nigel fraction, the intensities of the marker band of the membrane disorder (2855 and 2926 cm-^1^) decreased significantly from 0.364±0,020 and 0.544±0,026 in the absence of nigel extract) to 0.273±0.008 and 0.448±0.018 (in presence of Nigel extract), respectively. Even the νsCH_2_ to νasCH_2_ ratio decreased from 0.669±0.004 to 0.610±0.007. These results show that the Nigel fraction reestablished the normal synthesis of unsaturated fatty acid.

**Fig 5:**
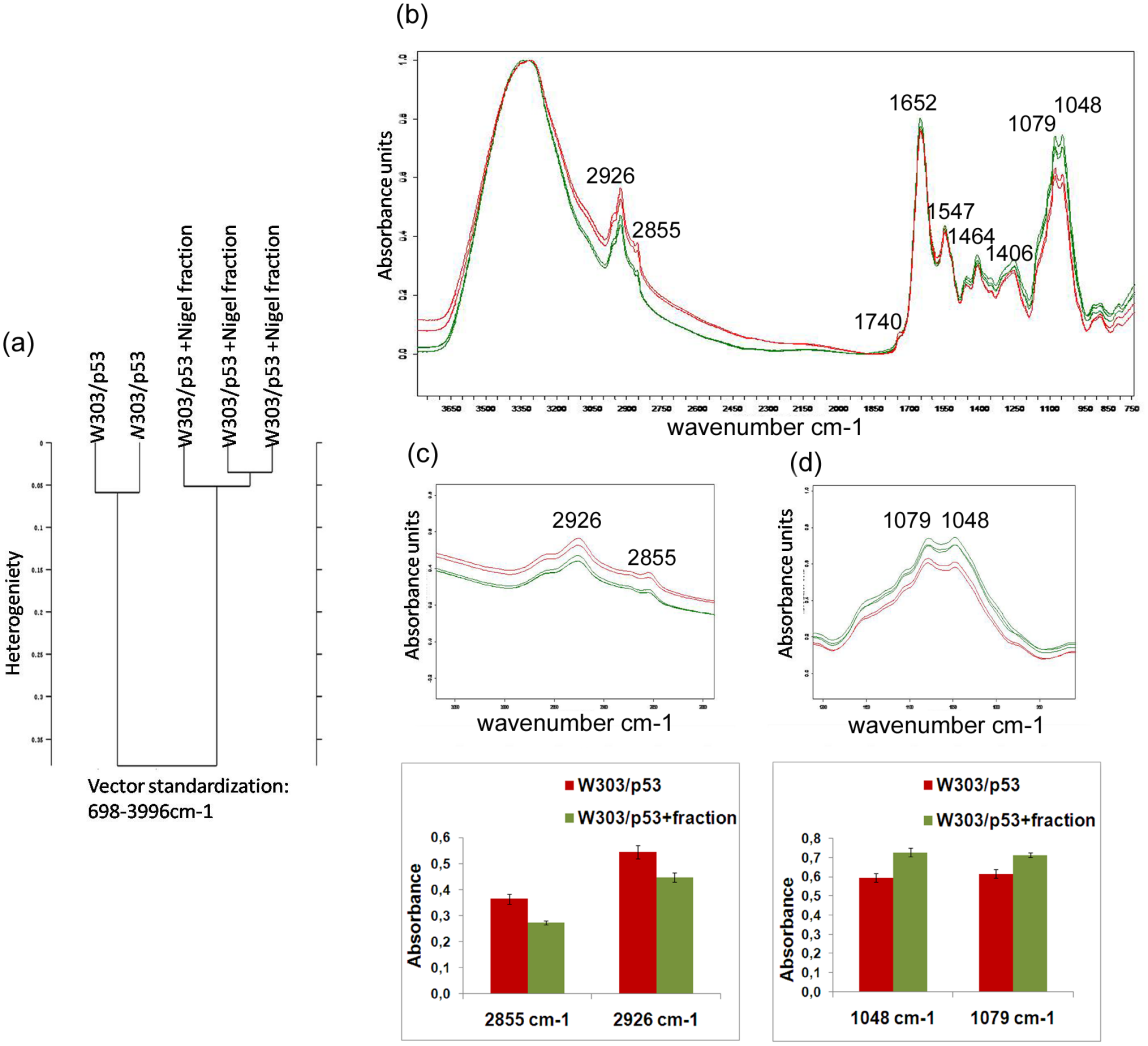
FTIR spectroscopy analysis of the whole recombinant yeast clones W303/p53 grown in MMGAL in absence and presence of purified Nigel extract. (a) Dendrogram representing the cluster analysis of W303/p53 grown in MMGAL in absence and in presence of nigel fraction and W303/p53. (b) FTIR spectra of W303/p53 (red line) and W303/p53 (green line) grown on MMGAL in absence and presence of nigel fraction, (c) spectral intensity band changes in 3000–2820cm−1 region showing membrane changes and (d) spectral intensity band changes in 1187-945cm-1 region showing DNA changes. The values are means of three independent experiments.

### 1187–945 cm^-1^ region

DNA fragmentation in W303/p53 cells was highlighted by the intensity decrease of the band 1079 and 1048 cm^-1^ being at 0.615±0.019 and 0.595±0.020 (Fig 5), respectively. In the same p53+ cells incubated with the Nigel fraction, these intensities reached similar values for the control W303/pDP, being at 0.715±0.023 and 0.716±0.023 respectively. Thus the Nigel fraction prevents the DNA fragmentation.

Moreover, νC = O stretching vibrations of the ester functional groups, observed at 1740 cm^-1^ in W303/p53 cultured in MMGAL, disappeared in the presence of the Nigel fraction, which means that this fraction inhibited the PS exposure.

## Discussion

The most typical markers of apoptosis are DNA fragmentation and phosphatidylserine externalization. Both markers are well conserved in yeast and are harnessed here to show that p53 overexpression induces apoptosis ([49]; this work). The present FTIR study of yeast apoptosis highlighted several intensity changes of bands that are characteristic of the same markers. These findings prove that the FTIR technique is a good tool to study the biochemical changes in yeast during apoptosis.

### Phospholipids changes

Conformational changes in 3000–2820cm^−1^ are reported in ethanol-stressed yeast cells [24] and can possibly be associated to biochemical changes resulting from the disorganization of the cell plasma membrane itself due to the increase of unsaturated fatty acids leading to an increase of the yeast membrane fluidity [63].

Increased intensities in this spectral area can be considered as an important apoptosis marker that was previously shown in many studies on mammalian cells indicating a conformational disorder in cell membrane [32,33,35,36,61]. It could also reflect the variation of lipid amounts around the nucleus, also known to increase in apoptosis [34].

The CH_2_-symmetric ratio to CH_2_-antisymetric stretching is another way to label the cell membrane morphology alterations [64]; in our study it increased significantly in W303/p53 cells that underwent apoptosis.

It has been reported that apoptotic cells change in morphology [65], consisting in plasma membrane pores, or leaky spots created by rigid patches of tightly packed phospholipids in the inner plasma membrane. These rigid patches are formed due to an increased amount of saturated fatty-acids [64] and increased membrane fluidity [65]. This spectral change could be correlated with the major exposure of PS in the outer leaflet of the plasma membrane [66].

### Protein changes

This originates from the vibrations of the amide groups (COONH) of proteins, and is often an indicator of their secondary structure. The vibrational band at~1652 cm^-1^ results from the stretching vibrations of C-O that is a characteristic of the α-helix structure [67]. However the 1547 cm^-1^ absorption band, which is a combination of the (N–H) bending and n(C–N) stretching vibrations, is attributed to the β-sheet secondary structure of proteins [68].

The amide bands were harnessed to study all the protein structures including the membrane proteins. Indeed, several studies described the association of an increase in β-sheet structures with apoptosis [34,36,38,69]. Caspari et al 2003 [37] suggested that conformational changes of protein structures might be correlated with proteolysis exerted by apoptotic caspases [70,71]. Differences in yeast cell composition after exposure to nanosilver revealed a significant downshift (20 cm−1) of the amide I peak at 1655 cm−1 and a loss in α-helix structures, thereby indicating the disordered secondary structures of proteins [72],

This conformational change allows the identification of apoptosis spectral marker that could be assigned to protein oxidation [73]. The PS is a negatively-charged phospholipid that is normally predominant in membrane leaflets facing the cytosol; the loss of the membrane phospholipid asymmetry (during apoptosis) results in the inversion of the PS exposure. Thus, the conformational disorder of ester stretching in PS, corresponding to the increase of C = O ester groups of phosphatidylserine, could be the signal of phospholipids alteration that increases the membrane permeability and fluidity [74]. In this study the phospholipids spectral changes substantiate the exposure of the PS at the outer surface of the plasma membrane induced by the p53 expression in yeast [49],

### Nucleic acids changes

DNA damages were expressed by the decrease of 1048 cm-1 and 1079 cm^-1^ absorption bands that reflect the cell DNA content [52] and indicate the DNA oxidative damage degree [25].

### Anti-apoptotic effect of *Nigella sativa* seeds

Given that the yeast has similarities with mammalian cells at the molecular and functional levels, it would contribute to understand some molecular mechanisms of higher eukaryotes such as apoptosis. In this study, p53+ yeast cells were used as a model for the screening of anti-apoptotic molecules. *Nigella sativa* extract was shown to prevent yeast apoptosis by following PS externalization and DNA fragmentation. In addition, there was no significant conformational changes between W303/pDP MMGAL and W303/pDP MMGAL+Nigel fraction. This means that *Nigella sativa* did not change the yeast metabolism nor its growth.

## Conclusion

This study confirms that FTIR spectroscopy technique is a very sensitive and useful tool for the detection and monitoring of biochemical changes of apoptosis mediated by p53 over-expression in yeast *S*. *cerevisiae*, previously analyzed by flow-cytometry. Moreover, highlighted apopotosis marker bands were used to evaluate the protective effect of *Nigella sativa* extract on p53-mediated apoptosis. This rapid and specific method could undoubtedly be used for a large screening of other natural biomolecules on yeast p53-mediated apoptosis.

## Abbreviations

FPLC: Fast-Performance Liquid Chromatography
FTIR: Fourier-transform infrared
HCA: Hierarchical cluster analysis
HPLC: High Performance Liquid Chromatography
MMGAL: Yeast minimal medium supplemented with 2% galactose
MMGLU: Yeast minimal medium supplemented with 2% glucose
PBS: Phosphate buffered saline
PI: propidium iodide
RID: refractive index detector
ROS: reactive oxygen species
SD: standard deviation
TUNEL: terminal-dUTP nick end labelling

## References

[1] Griffiths PR, De Haseth JA. Fourier Transform Infrared Spectrometry. John Wiley And Sons 1986; doi: 10.1002/bbpc.19860901224

[2] Erukhimovitch V, Karpasasa M, Huleihel M. Spectroscopic Detection And Identification Of Infected Cells With Herpes Viruses. Biopolymers. 2009; 91(1): 61–67. doi: 10.1002/bip.21082 18932269 10.1002/bip.21082

[3] Lasch P, Kneipp J. Biomedical Vibrational Spectroscopy. John Wiley and Sons inc; 2008: 79–104.

[4] Taillandie RE, Liquier F. infrared spectroscopy of DNA. Methods Enzymol. 1992; 211:307–335. 1406313 10.1016/0076-6879(92)11018-e

[5] Surewicz WK, Mantsch HH. New insight into protein secondary structure from resolution-enhanced infrared spectra. Biochi. Biophys Acta. 1988; 952(2): 115–130. . 3276352 10.1016/0167-4838(88)90107-0

[6] Mantsch HH, Mcelhaney R.N. phospholipid phase transitions in model and biological membranes as studied by infrared spectroscopy. Chem Phys.Lipids. 1991; 57(2–3); 213–226 2054905 10.1016/0009-3084(91)90077-o

[7] Choo LP, Jackson M, Halliday WC, Mantsch HH. infrared spectroscopy charactersisation of multiple sclerosis plaques in the human central nervous system. Biochim Biophys Acta. 1993; 1182(3): 333–337 8399370 10.1016/0925-4439(93)90078-f

[8] Nauman D, Helm D, Labischinski H. Microbiological characterizations by FT-IR spectroscopy. Nature 1991; 351(6321):81–2. doi: 10.1038/351081a0 1902911 10.1038/351081a0

[9] Helm D, Labischinski H, Challehn G, Naumann D. Classification and identification of bacteria by Fourier-transform infrared spectroscopy. J Gen Microbiol 1991(1); 137: 69–79 1710644 10.1099/00221287-137-1-69

[10] Dogan A, Ergen K, Budak F, Servecan F. evaluation Of Disseminated Candidiasis On An Experimental Model: A Fourrier Transform Infrared Study. Appl Spectrosc. 2007; 61(2): 199–203 17331312 10.1366/000370207779947459

[11] Cakmak G, Togan I, Servecan F. 17-beta-Estradiol Induced Compositional, Structural And Functional Changes In Rainbow Trout Liver Revealed By FT-IR Spectroscopy: A Comparative Study With Nonylphenol. Aquat Toxicol. 2006; 77(1): 53–63 16325934 10.1016/j.aquatox.2005.10.015

[12] Schultz CP, Liu KZ, Kerr PD, Mantsch HH. In Situ Infrared Histopathology Of Keratinization In Human Oral/Oropharyngeal Squamous Cell Carcinoma. Oncol Res. 1998; 10(5): 277–286 9802063

[13] Cheung HY, Cui J, Sun S. Real-time monitoring of Bacillus subtilis endospore components by Fourrier- transform infrared spectroscopy during germination. Microbiology. 1999; 145(5): 1043–1048 10376819 10.1099/13500872-145-5-1043

[14] Diem M, Boydston-White S, Chiriboga L. Infrared Spectroscopy Of Cells And Tissues: Shining Light Onto A Novel Subject. Appl Spectrosc. 1999; 53(4): 148–161

[15] Toyran N, Zorlu F, Severcan F. Effect Of Stereostatic Radiosurgery On Lipids And Proteins Of Normal And Hypoperfused Rat Brain Homogenates: A Fourrier Transform Infrared Spectroscopy Study. Int Jradiat Biol. 2005; 81(12): 911–8. doi: 10.1080/09553000600571022 10.1080/09553000600571022 16524846

[16] Zeroual W, Manfait M, Choisy C. FTIR spectroscopy study of perturbations induced by antibiotic on bacteria (Escherichia coli). Pathol Biol. 1995; 43(4): 300 7567119

[17] Portenier I, Waltimo T, Ørstavik D, Haapasal M. The susceptibility of starved stationary phase, and growing cells of Entorococcus faecalis to endodontic medicaments. J Endod. 2005; 31(5): 380–386. . 15851934 10.1097/01.don.0000145421.84121.c8

[18] Stehfest K, Toepel J, Wilhelm C. The application of micro-FTIR spectroscopy to analyze nutriment stress-related in biomass composition of phytoplankton algae Plant Physiol. Biochem. 2005; 43(7): 717–726. doi: 10.1016/j.plaphy.2005.07.001 10.1016/j.plaphy.2005.07.001 16122937

[19] Melin AM, Perromat A, Deleris G. sensitivity of deinococcus radiodurans to gamma-irradiation: a novel approach by Fourrier transform infrared spectroscopy. Arch Biochem Biophys. 2001; 394(2): 265 doi: 10.1006/abbi.2001.2533 11594741 10.1006/abbi.2001.2533

[20] Perromat A, Melin AM, Lorin C, Deleris G. Fourrier transform IR spectroscopic appraisal of radiation damage in Micrococcus luteus. Biopolymers. 2003; 72(4): 207 doi: 10.1002/bip.10381 12833474 10.1002/bip.10381

[21] Kamnev AA, Tugarova AV, Antonyuk LP, Tarantilis PA, Polissiou MG, Gardiner PH. Effects of heavy metals on plant-associated rhizobacteria: comparison of endophytic and non endophetic strains of Azospirillum brasilense. J Trace Elem Med Biol. 2005; 19(1): 91–95. doi: 10.1016/j.jtemb.2005.03.002 16240678 10.1016/j.jtemb.2005.03.002

[22] Moen B, Oust A, Langsrud O, Dorrell N, Marsden GL, Hinds J et al Multifactor approach for investigating global survival mechanisms of campylobacter jejuni under environmental condition. Appl Environ Microbiol. 2005; 71: 2086–2094. doi: 10.1128/AEM.71.4.2086-2094.2005 15812042 10.1128/AEM.71.4.2086-2094.2005 PMC1082531

[23] Mecozzi M, Pietroletti M, Di Mento R. Application of FTIR spectroscopy in ecotoxicological studies supported by multivariate analysis and 2D correlation spectroscopy. Vib Spectrosc. 2007; 44: 228–235. doi: 10.1016/j.vibspec.2006.11.006

[24] Szeghalmi A, Kaminskyj S, Gough KM. A Synchrotron FTIR microspectroscopy investigation of fungal hyphase grown under optimal and stressed conditions. Anal Bioanal Chem. 2007; 387: 1779–1789. doi: 10.1007/s00216-006-0850-2 17106657 10.1007/s00216-006-0850-2

[25] Corte L, Rellini P, Roscini L, Fatichenti F, Cardinali G. Development of a novel, FTIR (Fourrier Transform Infrared Spectroscopy) based, yeast bioassay for toxicity testing and stress response study. Anal Chim Acta. 2010; 659(1–2): 258–265. doi: 10.1016/j.aca.2009.11.035 20103133 10.1016/j.aca.2009.11.035

[26] Saharan RK, Sharma SC. FTIR spectroscopy and biochemical Investigation of ethanol stressed yeast Pachysolen tannophilus. Vib Spectrosc. 2011; 55: 85–89

[27] Bellisola G, Sorio C. Infrared Spectroscopy And Microscopy In Cancer Research And Diagnosis. Am J Cancer Res. 2012; 2(1): 1–21. 22206042 PMC3236568

[28] Wyllie AH. Glucocorticoid-Induced Thymocyte Apoptosis Is Associated With Endogenous Endonuclease Activation. Nature. 1980; 284(5756): 555–6. . 6245367 10.1038/284555a0

[29] Scott CE, Adebodun F. 13C-NMR Investigation of Protein Synthesis During Apoptosis In Human Leukemic Cell Lines. J Cell Physiol. 1999; 18: 1147–52. 10.1002/(SICI)1097-4652(199910)181:1<147::AID-JCP15>3.0.CO;2-M 10457362

[30] Gao Y, Huo X, Dong L, Sun X, Sai H, Wei G et al Fourier transform infrared microspectroscopy monitoring of 5-fluorouracil-induced apoptosis in SW620 colon cancer cells. Mol Med Reports. 2015; 11(4); 2585–2591. doi: 10.3892/mmr.2014.3088 25503826 10.3892/mmr.2014.3088 PMC4337715

[31] Kendall C, Isabelle M, Basant-Hegemark F, Hutchings J, Orr L, Barbarah J et al Vibrational Spectroscopy: A Clinical Tool For Cancer Diagnostics. Analyst. 2009; 134: 1029–1045. doi: 10.1039/b822130h 19475128 10.1039/b822130h

[32] Liu KZ, Shi MH, Mantsch HH. Molecular And Chemical Characterization Of Blood Cells By Infrared Spectroscopy: A New Optical Tool In Hematology. Blood Cells Mol Dis. 2005; 35(3): 404–12. doi: 10.1016/j.bcmd.2005.06.009 16126419 10.1016/j.bcmd.2005.06.009

[33] Liu KZ, Xu M, Scott DA. Biomolecular Characterisation Of Leucocytes By Infrared Spectroscopy. Br J Haematol. 2007; 136(5): 713–22. doi: 10.1111/j.1365-2141.2006.06474.x 17338777 10.1111/j.1365-2141.2006.06474.x

[34] Zhou J, Wang Z, Sun S, Liu M, Zhang H. A rapid method for detecting conformational changes during differentiation and apoptosis of HL60 cells by Fouriertransform infrared spectroscopy. Biotechnol Appl Biochem. 2001; 33(pt2): 127–132. . 11277866 10.1042/ba20000074

[35] Gaudenzi AS, Pozzi BD, Toroa P, Silvestri BI, Morrone BS, Castellano CAC. Cell Apoptosis Specific Marker Found By Fourier Transform Infrared Spectroscopy Spectroscopy Spectrosc. 2004; 18(3): 415–422. doi: 10.1155/2004/483591

[36] Jamin N, Miller L, Moncuit J, Fridman WH, Dumas P, Teillaud JL Chemical Heterogeneity In Cell Death: Combined Synchrotron IR And Fluorescence Microscopy Studies Of Single Apoptotic And Necrotic Cells. Biopolymers. 2003; 72(5): 366–373. doi: 10.1002/bip.10435 12949827 10.1002/bip.10435

[37] Gasparri F, Muzio M. Monitoring Of Apoptosis Of HL60 Cells By Fourier-Transform Infrared Spectroscopy. Biochem J. 2003; 369(pt2): 239–248. doi: 10.1042/BJ20021021 12383054 10.1042/BJ20021021 PMC1223092

[38] Zelig U, Kapelushnik J, Moreh R, Mordechai S, Nathan I. Diagnosis Of Cell Death By Means Of Infrared Spectroscopy. Biophys J. 2009; 97(7): 2107–2114. doi: 10.1016/j.bpj.2009.07.026 19804743 10.1016/j.bpj.2009.07.026 PMC2756387

[39] Madeo F, Frohlich E, Frohlich KU. A Yeast Mutant Showing Diagnostic Markers of Early and Late Apoptosis. J Cell Biol. 1997; 139(3): 729–734. 9348289 10.1083/jcb.139.3.729 PMC2141703

[40] Ludovico P, Sousa MJ, Silva MT, Leao C, Corte-Real M. Saccharomyces cerevisiae commits to a programmed cell death process in response to acetic acid. Microbiol. 2001; 147(pt9): 2409–2415. doi: 10.1099/00221287-147-9-2409 11535781 10.1099/00221287-147-9-2409

[41] Granot D, Levine A, Dor-Hefetz E. Sugar-induced apoptosis in yeast cells. FEMS Yeast Res. 2003; 4(1): 7–13. . 14554192 10.1016/S1567-1356(03)00154-5

[42] Madeo F, Frohlich E, Ligr M, Grey M, Sigrist SJ, Wolf DH et al Oxygen stress: a regulator of apoptosis in yeast. J Cell Biol. 1999; 145(4): 757–767. . 10330404 10.1083/jcb.145.4.757 PMC2133192

[43] Balzan R, Sapienza K, Galea DR, Vassallo N, Frey H, Bannister VH. Aspirin commits yeast cells to apoptosis depending on carbon source. Microbiol. 2004; 150(pt1): 109–115. doi: 10.1099/mic.0.26578-0 10.1099/mic.0.26578-0 14702403

[44] Ferreira P, Cardoso T, Ferreira F, Fernandes-Ferreira M, Piper P, Sousa MJ. Mentha piperita essential oil induces apoptosis in yeast associated with both cytosolic and mitochondrial ROS-mediated damage. FEMS Yeast Res. 2014 doi: 10.1111/1567-1364.12189 25065265 10.1111/1567-1364.12189

[45] Ligr M, Madeo F, Frohlich E, Hilt W, Frohlich KU, Wolf DH. Mammalian Bax triggers apoptotic changes in yeast. FEBS Lett. 1998; 438(1–2): 61–65. doi: 10.1016/S0014-5793(98)01227-7 9821959 10.1016/s0014-5793(98)01227-7

[46] Ryser S, Vial E, Magnenat E, Schlegel W, Maundrell K. Reconstitution of caspase-mediated cell-death signaling in *Schizosaccharomyces pombe* Curr. Genet. 1999; 36(1–2): 21–28. 10.1007/s002940050468 10447591

[47] Carmona-Gutierrez D, Eisenberg T, Büttner S, Meisinger C, Kroemer G, Madeo F. Apoptosis In Yeast: Triggers, Pathways, Subroutines, Cell Death Differ. 2010; 17(5): 763–773. doi: 10.1038/cdd.2009.219 20075938 10.1038/cdd.2009.219

[48] Madeo F. Herker E, Maldener C, Wissing S, Lachelt S, Herlan M et al A Caspase-Related Protease Regulates Apoptosis In Yeast. Mol Cell. 2002; 9(4): 911–917. . 11983181 10.1016/s1097-2765(02)00501-4

[49] Yacoubi-Hadj Amor I, Smaoui K, Chaabene I, Mabrouk I, Djemal L, Elleuch H A et al Human P53 Induces Cell Death And Down Regulates Thioredoxin Expression In Saccharomyces Cerevisiae. FEMS Yeast Res 2008; 8(8): 1–9. doi: 10.1111/j.1567-1364.2008.00445.x 19054132 10.1111/j.1567-1364.2008.00445.x

[50] Thomas BJ, Rothstein R. Elevated recombination rates in transcriptionally active DNA. Cell. 1989; 56(4): 619–630. . 2645056 10.1016/0092-8674(89)90584-9

[51] Cullin C, Pompon D. Synthesis of functional mouse cytochromes P-450 P1 and chimeric P-450 P3-1 in the yeast Saccharomyces cerevisiae, Gene. 1988; 65(2): 203–217. 3044926 10.1016/0378-1119(88)90457-x

[52] Mokdad-Gargouri R, Belhadj K, Gargouri A. Translational control of human p53 expression in yeast mediated by 50-UTR-ORF structural interaction, Nucleic Acids Res. 2001; 29(5): 1222–1227. 11222773 10.1093/nar/29.5.1222 PMC29724

[53] Madeo F, Frohlich E & Frohlich KU (1997) A yeast mutant showing diagnostic markers of early and late apoptosis. J Cell Biol 139(3): 729–734. 9348289 10.1083/jcb.139.3.729 PMC2141703

[54] Amiel C, Mariey L, Curk-Daubié MC, Pichon P, Travert J. Potentiality Of Fourier Transform Infrared Spectroscopy (FTIR) For Discrimination And Identification Of Dairy Lactic Acid Bacteria. Lait 2000; 80(3): 1–15.

[55] Guibet M, Kervarec N, Génicot S, Chevolot Y, Helbert W. Complete assignment of 1H and 13C NMR spectra of Gigartina skottsbergii lambda-carrageenan using carrabiose oligosaccharides prepared by enzymatic hydrolysis. Carbohydr Res. 2006; 341(11): 1859–69. doi: 10.1016/j.carres.2006.04.018 16716278 10.1016/j.carres.2006.04.018

[56] Saeed Mahmood M, Gilani AH, Afsheen K, Ayesha R, Ashfaq MK. The In Vitro Effect of Aqueous Extract of Nigella sativa Seeds on Nitric Oxide Production, Phytother. Res. 2003; 17(8): 921–924. doi: 10.1002/ptr.1251 13680825 10.1002/ptr.1251

[57] Sambrook J, Fritsch EF, Maniatis T. Molecular cloning, a laboratory manual. Cold Spring Harbor Laboratory Press.

[58] Yu C, Irudayaraj J. spectroscopic characterization of microorganisms by fourrier transform infrared microspectroscopy. Biopolymers. 2005; 77(6): 368–377. doi: 10.1002/bip.20247 15700299 10.1002/bip.20247

[59] Gautam R, Chandrasekar B, Deobagkar-Lele M, Rakshit S, Kumar BN V, Umapathy S et al Microspectroscopy. Identification Of Early Biomarkers During Acetaminophen-Induced Hepatotoxicity By Fourier Transform Infrared Microspectroscopy. Plos One. 2012; 7(9): E45521 doi: 10.1371/journal.pone.0045521 23029070 10.1371/journal.pone.0045521 PMC3446881

[60] Benedetti E, Teodori L, Trinca ML, Vergamini P, Salvati F, Mauro F et al New approach to the study of human solid tumor cells by means of FT-IRmicrospectroscopy,Appl. Spectrom. 1990; 44(8): 1276–1280

[61] Liu K.-Z, Mantsch HH. Apoptosis-induced structural changes in leukemia cells identified by IR spectroscopy. J Mol Struct 2001; 565-566(1–3): 299–304. doi: 10.1016/S0022-2860(00)00817-6

[62] Benedetti E, Palatresi MP, Vergamini P, Papineschi F, Andreucci MC, Spremolla G. Infrared characterization of nuclei isolated from normal and leukemic (B-CLL) lymphocytes: part III. Appl Spectrom. 1985; 40: 39–43

[63] Sajbidor J, Ciesarova Z, Smogrovicova D. influence of ethanol on the lipidcontent and fatty acid composition of saccharomyces cerevisiae. Folia Microbiol. 1995; 40(5): 508–510. 8763146 10.1007/BF02814733

[64] Van Den Drieschea S, Iulianob F, Haidenc C, Pucciarelli D, Breitenederd DH, Pastorekovab S et al Cell Membrane Morphology Analysis Using An Infrared Sensor System. Sensors And Actuators B. 2013; 179: 150–156. doi: 10.1016/j.snb.2012.10.139

[65] Singh JK, Dasgupta A, Adayev T, Shahmehdi SA, Hammond D, Banerjee P. Apoptosis Is Associated With An Increase In Saturated Fatty Acid Containing Phospholipids In The Neuronal Cell Line, HN2-5. Biochim Biophy Acta. 1996; 1304(3): 171–178. doi: 10.1016/S0005-2760(96)00134-8 10.1016/s0005-2760(96)00134-8 8982263

[66] Pozzi D, Grimaldi P, Gaudenzi S, Di Giambattista L, Silvestri I, Morrone S et al UVB-Radiation-Induced Apoptosis In Jurkat Cells. A Coordinated Fourrier Transform Infrared Spectroscopy Study. Rad Res. 2007; 168(6): 698–705. doi: 10.1667/RR0991.1 18088183 10.1667/RR0991.1

[67] Abbott GW, Ramesh B, Srai SK. Interaction Between Soluble And Membrane-Embedded Potassium Channel Peptides Monitored By Fourier Transform Infrared Spectroscopy. Plos One. 2012; 7: E49070 doi: 10.1371/journal.pone.0049070 23145073 10.1371/journal.pone.0049070 PMC3493504

[68] Mantsch HH, Choo-Smith LPi, Shaw RA. Vibrational Spectroscopy And Medicine: An Alliance In The Making. Vib Spectrosc. 2002; 30(1): 31–41.

[69] Gault N, Lefaix J. Infrared Microspectroscopic Characteristics Of Radiation-Induced Apoptosis In Human Lymphocytes. Radiat Res. 2003; 160(2): 238–250. 12859236 10.1667/rr3020.1

[70] Casiano CA, Ochs RL, Tan EM. Distinct Cleavage Products Of Nuclear Proteins In Apoptosis And Necrosis Revealed By Autoantibody Probes. Cell Death Differ. 1998; 5: 183–190 doi: 10.1038/sj.cdd.4400336 10200463 10.1038/sj.cdd.4400336

[71] Dixit VM. Role of ICE-Proteases In Apoptosis. Adv Exp Med Biol. 1996; 406: 113–117. . 8910676 10.1007/978-1-4899-0274-0_11

[72] Saulou C, Jamme F, Maranges C, Fourquaux I, Despax B, Raynaud P et al Synchrotron FTIR microspectroscopy of the yeast Saccharomyces cerevisiae after exposure to plasma-deposited nanosilver-containing coating Anal Bioanal Chem. 2010; 396(4):1441–1450. doi: 10.1007/s00216-009-3316-5 20012742 10.1007/s00216-009-3316-5

[73] Jamin N, Dumas P, Moncuit J, Fridman WH, Teillaud JL, Carr GL et al Highly Resolved Chemical Imaging of Living Cells by using Synchrotron Infrared Microspectrometry. Proc Natl Acad Sci 1998; 95(9): 4837 9560189 10.1073/pnas.95.9.4837 PMC20174

[74] Wu BB, Gony YP, Wu XH, Chen YY, Chen FF, Jin LT et al Fourrier Transform Infrared Spectroscopy For The Distinction Of MCF-7 Cells Treated With Different Concentration Of 5-Fluorouracil. Journal of Translational Medicine 2015; 13: 1–8. 25884618 10.1186/s12967-015-0468-2 PMC4391530

